# The Expanding Cell Diversity of the Brain Vasculature

**DOI:** 10.3389/fphys.2020.600767

**Published:** 2020-12-03

**Authors:** Jayden M. Ross, Chang Kim, Denise Allen, Elizabeth E. Crouch, Kazim Narsinh, Daniel L. Cooke, Adib A. Abla, Tomasz J. Nowakowski, Ethan A. Winkler

**Affiliations:** ^1^Department of Neurological Surgery, University of California, San Francisco, San Francisco, CA, United States; ^2^Department of Anatomy, University of California, San Francisco, San Francisco, CA, United States; ^3^Department of Psychiatry and Behavioral Sciences, University of California, San Francisco, San Francisco, CA, United States; ^4^The Eli and Edythe Broad Center for Regeneration Medicine and Stem Cell Research, University of California, San Francisco, San Francisco, CA, United States; ^5^Department of Pediatrics, University of California, San Francisco, San Francisco, CA, United States; ^6^Department of Radiology, University of California, San Francisco, San Francisco, CA, United States; ^7^Chan Zuckerberg Biohub, San Francisco, CA, United States

**Keywords:** neurovascular unit, single cell sequencing, endothelial cells, pericytes and vascular smooth muscle cells, perivascular macrophages, perivascular fibroblasts, astrocytes, blood brain barrier

## Abstract

The cerebrovasculature is essential to brain health and is tasked with ensuring adequate delivery of oxygen and metabolic precursors to ensure normal neurologic function. This is coordinated through a dynamic, multi-directional cellular interplay between vascular, neuronal, and glial cells. Molecular exchanges across the blood–brain barrier or the close matching of regional blood flow with brain activation are not uniformly assigned to arteries, capillaries, and veins. Evidence has supported functional segmentation of the brain vasculature. This is achieved in part through morphologic or transcriptional heterogeneity of brain vascular cells—including endothelium, pericytes, and vascular smooth muscle. Advances with single cell genomic technologies have shown increasing cell complexity of the brain vasculature identifying previously unknown cell types and further subclassifying transcriptional diversity in cardinal vascular cell types. Cell-type specific molecular transitions or zonations have been identified. In this review, we summarize emerging evidence for the expanding vascular cell diversity in the brain and how this may provide a cellular basis for functional segmentation along the arterial-venous axis.

## Introduction

Continued expansion and sophistication of the mammalian brain has resulted in an astonishing level of cell diversity (Zeisel et al., 2015, 2018; Neubauer et al., 2018; Saunders et al., 2018; Tasic et al., 2018; Cadwell et al., 2019; Hodge et al., 2019). It is believed that this cell diversity helps provide the sub-specialization of cellular function required to face the everchanging contexts of life—such as brain development, aging, and responses to injury or environmental insults. Despite this sophistication, the brain remains vitally dependent on the cerebrovasculature for metabolic exchange with circulating blood—including the delivery of oxygen, glucose, and other metabolites and clearance of toxic metabolic by-products (Winkler et al., 2014a, 2015; Iadecola, 2017; Sweeney et al., 2018). Disruption of the cerebral circulation for even minutes may have profound neurologic implications (Campbell et al., 2019). To meet dynamic metabolic needs, the cerebrovasculature has developed a highly evolved blood–brain barrier (BBB) to permit regulated molecular transport while preventing influx of circulating blood cells or potentially toxic pathogens or plasma proteins (Zhao et al., 2015; Sweeney et al., 2019). Coordinated communication between neurons, glial, and vascular cells facilitates local regulation of cerebral blood flow (CBF) to ensure blood supply is tightly matched with metabolic demand—a process known as “neurovascular coupling” (Uhlirova et al., 2016; Iadecola, 2017).

Over the past two decades, increased recognition that interconnection between multiple cell types is essential for brain vascular function and has resulted in advancement of the concept of the “neurovascular unit” (Iadecola, 2017; Sweeney et al., 2018). Much of this work, however, has focused on cardinal cell types without acknowledgment of further sub-specialization or vascular cellular diversity. Development of single cell RNA sequencing (scRNAseq) technologies have now allowed unbiased characterization of transcriptional heterogeneity in brain vascular cells (Zeisel et al., 2015, 2018; Sabbagh et al., 2018; Saunders et al., 2018; Vanlandewijck et al., 2018). While heterogeneity in glial and neurons have been reviewed elsewhere (Miller, 2018; Cembrowski and Spruston, 2019; Masuda et al., 2020), we summarize the emerging evidence of added cell diversity within brain vascular cells highlighting how cellular sub-specialization contributes to regionalized vascular function along the arterial-venous axis.

### Cardinal Cell Types of the Cerebrovasculature

**FIGURE 1:**
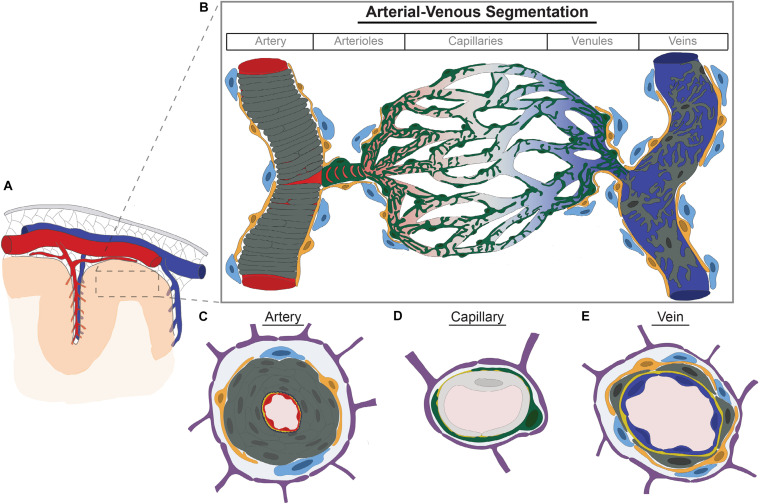
Brain vascular cell diversity along the arterial-venous axis. **(A)** Large arteries (red) and cortical veins (blue) course within the subarachnoid space (gray mesh) inside the dura mater (gray). Cerebral arteries course along the brain’s pial surface and within sulci giving rise to pial and penetrating arterioles which pierce the brain parenchyma. **(B)** Arterioles progressively branch to form capillaries. Capillaries then converge to form venules which converge further to form veins. Each vessel segment along the arterial-venous axis are comprised of different cell types—including endothelium (shown in red, gray, and blue to denote transcriptional zonations), vascular smooth muscle cells (dark gray, vSMCs), pericytes (green), perivascular fibroblasts (yellow), and perivascular macrophages (light blue). Pericytes further adopt different morphologic configurations along the arteriovenous axis, such as ensheathing pericytes (pre-capillary arterioles) and mesh-like and stellate pericytes in more distal arterioles, capillaries, and venules. vSMCs form concentric rings in arteries but are discrete and stellate shaped in veins. Perivascular fibroblasts and macrophages are found in arteries, arterioles, venules, and veins, but not capillaries. **(C–E)** Cross section of cellular components of artery **(C)**, capillary **(D)**, and veins **(E)**. Endothelium depicted in artery, capillary, and vein are shown in red, light gray, and blue, respectively, to denote transcriptional differences or molecular zonations defined and validated by independent groups with single cell RNA-sequencing. Dark gray, vascular smooth muscle cells; Navy blue, internal elastic lamina; Green, pericytes; Orange, perivascular fibroblasts; Light blue, perivascular macrophages; Yellow, basement membrane; Purple, astrocyte end feet.

The cerebrovasculature consists of multiple cell types—including endothelial cells (ECs), pericytes (PCs), and vascular smooth muscle cells (vSMCs) (Winkler et al., 2011, 2019; Zhao et al., 2015; Iadecola, 2017; Sweeney et al., 2018; Figure 1). Newly defined perivascular macrophages (PVMs) and perivascular fibroblast-like cells (PVFBs) also reside along the vascular wall (Zeisel et al., 2015; Saunders et al., 2018; Vanlandewijck et al., 2018). Astrocyte foot processes wrap around the vessel wall and create the Virchow Robin space—a perivascular space important for brain interstitial fluid and cerebrospinal fluid exchange known as the “glymphatic system” (Wardlaw et al., 2020). Parenchymal glial or neuronal cell bodies are closely apposed to the brain vasculature and rarely exceed > 15 μm from each vessel (Tsai et al., 2009). Here, we summarize established characteristics of vascular cells and briefly highlight their brain-specific features and functions.

#### Endothelial Cells

Blood vessels are made up of a single layer of ECs that form a tubular structure (Zhao et al., 2015; Sweeney et al., 2019). The blood facing surface, or luminal surface, is covered by the glycocalyx—a gel-like covering comprised of glycoproteins, proteoglycans, and glycosaminoglycans (Ando et al., 2018; Kutuzov et al., 2018). Most brain ECs have a continuous cell membrane which lacks pores or fenestrations. Adjacent ECs are securely connected to one another by adherens junctional protein complexes and tight junction protein complexes occludes the intercellular cleft. Brain ECs have a high density of tight junction proteins which precludes significant paracellular flow and is a key feature regulating size-restricted transcellular passage of molecules (Zhao et al., 2015; Sweeney et al., 2019). Unlike other organs, ECs also display lower rates of pinocytosis or non-specific bulk-flow vesicular transport (Ben-Zvi et al., 2014; Andreone et al., 2017; Chow and Gu, 2017). The brain-facing or abluminal EC membrane is embedded in a protein-rich basement membrane (BM) serving as a cellular scaffolding essential for interactions with neighboring cells (Thomsen et al., 2017).

Low rates of pinocytosis and membrane permeability make brain ECs the anatomic site of the BBB. The BBB permits gases, e.g., carbon dioxide and oxygen, and lipophilic and small molecules (< 400 Da) to freely enter the brain, but limits circulating cells and other molecules from crossing the endothelium without regulated transport systems (Banks, 2009; Sweeney et al., 2019). ECs contain a number of different transmembrane transporters to regulate influx of circulating nutrients and proteins—including carrier-mediated, receptor-mediated, and active transporter proteins (Sweeney et al., 2019; Tjakra et al., 2019). Active efflux transporters or other transport proteins also help remove potentially toxic waste products from brain into the blood (Hladky and Barrand, 2018). ECs sense shear stress from blood flow or receive signaling cues from neurons or glia to secrete vasoactive substances to help modulate vasomotor responses in adjacent mural cells and titrate local blood flow (Chen et al., 2014; Iadecola, 2017; Chow et al., 2020).

#### Pericytes

In the microvasculature, e.g., capillaries, venules, and select arterioles, PCs are embedded in the shared BM and extend foot processes which cover much of the vessel wall (Winkler et al., 2012, 2014a; Grant et al., 2019). Unlike vSMCs which surround blood vessels circumferentially, PCs generally extend thin longitudinal processes with further delicate elaborations to cover more of the vascular surface. Interestingly, PCs cover the vasculature in a mutually exclusive pattern and do not overlap; upon experimental ablation of one pericyte, the neighboring pericytes will then grow their surface area to take over the newly absent territory (Berthiaume et al., 2018). Pericytes contribute to endothelial BBB properties through upregulation of tight junctional protein complexes and/or downregulation of transcytotic pathways—such as those mediated by plasmalemma vesicle associated protein (*Plvap*) or caveolin-1 (Hellstrom et al., 2001; Armulik et al., 2010; Bell et al., 2010; Daneman et al., 2010). They also secrete BM proteins to stabilize vessels (Thomsen et al., 2017). To further regulate brain molecular exchanges, PCs are enriched in multiple carrier- and receptor-mediated transporters, active transporter proteins, or ion channels (He et al., 2016; Vanlandewijck et al., 2018; Sweeney et al., 2019).

PCs modulate capillary diameter in response to neuronal or astrocytic cues (Peppiatt et al., 2006; Hall et al., 2014; Cai et al., 2018), but the functional significance of this is unclear. Some have shown that PCs regulate local CBF regulation and neurovascular coupling (Kisler et al., 2017b, 2020). Others have found this is principally the role of vSMCs, such as those in arterioles, or pre-capillary sphincters (Fernandez-Klett et al., 2010; Hill et al., 2015; Grubb et al., 2020). This discrepancy likely stems from a lack of clarity in regards to the exact definition of vSMCs vs. PCs. PCs supply trophic support to neurons and also modulate immune responses (Rustenhoven et al., 2017; Nikolakopoulou et al., 2019; Uemura et al., 2020). For example, PCs modulate expression of endothelial cell adhesion molecules, such as intercellular adhesion molecule-1 (*Icam1*), and may also play a direct role in the phagocytosis of extracellular proteins (Bell et al., 2010; Sagare et al., 2013; Ma et al., 2018). This has been suggested to increase after brain injury, such as ischemic stroke, and pericytes may assume a microglial-like phenotype (Ozen et al., 2014; Sakuma et al., 2016). Others have suggested pericytes may serve as multi-potent stem cells *in vitro* (Dore-Duffy et al., 2006; Crisan et al., 2008; Karow et al., 2018). However, lineage tracing has shown that pericytes do not significantly contribute to other cell lineages *in vivo* and do not differentiate into microglia following acute brain injuries (Guimaraes-Camboa et al., 2017; Huang et al., 2020).

#### Vascular Smooth Muscle Cells

vSMCs from concentric rings in larger arteries and become less layered and more sparse as vessels progressively branch to form pial and penetrating arterioles (Iadecola, 2017; Frosen and Joutel, 2018). In veins, vSMCs remain as discrete cells. Due to their location and composition, vSMCs contribute much of structural stability to the vessel wall and mediate synthesis and turnover of extracellular matrix proteins, such as collagen and elastin (Iadecola, 2017; Frosen and Joutel, 2018).

vSMCs serve as contractile cells and express a number of contractile proteins or associated regulatory proteins, such as smooth muscle alpha actin (*ACTA2*), and receptors to numerous vasoactive molecules, including adenosine, prostaglandins, or catecholamines, and to myogenic or flow-related stimuli (Koller and Toth, 2012; He et al., 2016; Kisler et al., 2017a; Chasseigneaux et al., 2018). Through these contractile properties, vSMCs contribute to regulation of cerebral blood flow and autoregulation (Fernandez-Klett et al., 2010; Hill et al., 2015; Iadecola, 2017). Arterial pulsations and/or oscillations in relaxation or contraction of vSMCs contribute to perivascular fluid flow via the glymphatic system (Iliff et al., 2013; Aldea et al., 2019; Wardlaw et al., 2020). Studies have supported that arteriolar vSMCs are the predominate site of regional CBF regulation (Hill et al., 2015; Grutzendler and Nedergaard, 2019), but the relative contributions of vSMCs and PCs to neurovascular coupling continues to be debated (Hamilton et al., 2010; Hall et al., 2014; Hill et al., 2015; Kisler et al., 2017b; Winkler et al., 2017).

#### Perivascular Fibroblast-Like Cells

A recent constellation of unbiased scRNAseq studies have identified PVFBs on all vessels except capillaries (Marques et al., 2016; Saunders et al., 2018; Vanlandewijck et al., 2018; Zeisel et al., 2018). PVFBs are located within the Virchow-Robin space and loosely adhere to vessels through small, fine cellular processes (Soderblom et al., 2013; Vanlandewijck et al., 2018; Rajan et al., 2020). PVFBs express platelet-derived growth factor receptor-α (*Pdgfr*α) and a number of extracellular matrix proteins—including fibrillar and non-fibrillar collagens, collagen-modifying enzymes, and small leucin-rich proteoglycans, such as lumican (*lum*) and decorin (*Dcn*) (Vanlandewijck et al., 2018; Rajan et al., 2020). The function of these new-defined cells has not been completely characterized. Emerging evidence early in zebrafish development in other vascular beds, such as intersegmental vessels, has suggested that PVFBs stabilize the vasculature prior to appearance of PCs, and lineage tracing has suggested a subpopulation of PVFBs may serve as PC progenitor cells (Rajan et al., 2020). In the mouse brain, however, collagen type 1 alpha 1 chain (*Col1a1*)-positive PVFBs are not detected until after the birth (Kelly et al., 2016). Therefore, it is presently unclear as to the role if any PVFBs play in the developing or adult mammalian brain vasculature under physiologic conditions. In response to traumatic injury, PVFBs have been suggested to contribute to scar formation (Soderblom et al., 2013). Others have ascribed scar formation to a subpopulation of PCs which may highlight difficulties in distinguishing perivascular cell types (Goritz et al., 2011; Dias et al., 2018).

#### Perivascular Macrophages

PVMs are myeloid cells distinct from microglia that reside in the Virchow-Robin space surrounding arterioles and venules (Lapenna et al., 2018; Yang et al., 2019). PVMs serve a vital role in the phagocytosis of blood-borne pathogens and antigen presentation to induce protective immune responses (Lapenna et al., 2018; Kierdorf et al., 2019). They also limit EC production of pro-inflammatory mediators suggesting a dual regulatory role in immune response (Serrats et al., 2010). PVMs promote EC BBB properties and tight junction protein expression (Zenker et al., 2003). Others have shown that PVMS modulate EC expression of nutrient receptors—such as solute carrier family 2 member 1 (*Slc2a*)—playing a homeostatic role in cerebral glucose metabolism (Jais et al., 2016). In brain regions devoid of a BBB, such as the circumventricular organs, PVM uptake restricts influx of blood-borne proteins and macromolecules providing a size selective barrier (Willis et al., 2007). Phagocytic properties are not limited to blood-borne molecules and may help clear other extracellular proteins, such as amyloid-β (Hawkes and McLaurin, 2009).

#### Astrocytes

Brain vascular cells are ensheathed by astrocyte end feet which form the outer limit of the perivascular Virchow-Robin space (Iliff et al., 2012; Rasmussen et al., 2018). Adjacent astrocyte end feet are connected via both tight and gap junctions which create a semi-permeable barrier—known as the glia limitans (Horng et al., 2017; Kutuzov et al., 2018). This secondary barrier further restricts radial diffusion of large solutes and circulating inflammatory cells (Nuriya et al., 2013; Horng et al., 2017; Kutuzov et al., 2018). Expression of transmembrane pores within astrocyte end feet, e.g., aquaporin-4, or ion pumps, e.g., Kir 4.1 potassium channels, regulate molecular exchanges between cerebrospinal fluid (CSF) within the perivascular space and brain interstitial fluid underlying the “glymphatic system” (Iliff et al., 2012; Rasmussen et al., 2018; Wardlaw et al., 2020). Interactions with adjacent PCs help maintain astrocyte end foot polarity and transport systems (Armulik et al., 2010; Gundersen et al., 2014). Astrocytes also contribute directly to EC BBB properties and secrete BM proteins, such as laminin (Abbott et al., 2006; Yao et al., 2014).

Astrocytes express metabotropic glutamate receptors (mGluR) and purinergic receptors (P2YR) which detect neuronal by-products of neuronal activation, such as glutamate or adenosine triphosphate, which help trigger section of vasoactive molecules—including arachidonic acid, associated derivatives, and prostaglandin E2 (Abbott et al., 2006; Iadecola, 2017; Kisler et al., 2017a). Low frequency stimuli or resting state fluctuations in neuronal activity have been shown to not provoke calcium signaling in astrocytes (Gu et al., 2018). Thus, it has been hypothesized that slow hemodynamic fluctuations in blood flow are driven largely by neurons and astrocytes play a modulating, but not triggering role in neurovascular coupling (Gu et al., 2018).

### Variations in Cytoarchitecture and Cell Morphology

Large muscular arteries, such as the internal carotid arteries and vertebral arteries which coalesce to form the basilar artery, enter the subarachnoid space and form a complex anastomotic loop known as the “circle of Willis.” Large cerebral arteries course along the brain’s pial surface tapering and branching giving rise to pial and penetrating arterioles. Arterioles branch to give rise to an immense capillary tree. Capillaries ultimately converge to form venules which further converge to form veins. Large cortical veins within the subarachnoid space ultimately connect to venous sinuses contained within the dura mater to facilitate egress of blood from the brain. In the ensuing subsection, we describe the diversity in cell morphology of each cell type along these vascular transitions or regional variations when described in the central nervous system (summarized in Figure 1).

#### Endothelial Cells

For most brain regions, the EC membrane remains continuous without interruption (Zhao et al., 2015; Sweeney et al., 2019). Expression of the tight junction proteins remains constant throughout the arterial-venous axis in the brain, but show some ultrastructural differences—such as a greater overlap in arteries (Hanske et al., 2017). Higher permeability and reduced tight junction protein expression is reported for select CNS regions—such as the spinal cord (Winkler et al., 2012). In brain circumventricular organs—such as the subfornical organ, organum vasculosum of the lamina terminalis and area postrema—or choroid-plexus, ECs contain fenestrations or intracellular pores which permit high permeability (Ghersi-Egea et al., 2018).

Preservation of BBB integrity also occurs through suppression of caveolae and transcytosis (Ben-Zvi et al., 2014; Andreone et al., 2017; Chow and Gu, 2017). Classically low rates of vesicular transport were thought to be uniform. However, higher rates of vesicular transport have been observed in arteries/arterioles whereas receptor-mediated transcytosis is primarily detected in post-capillary venules (Hanske et al., 2017; Kucharz et al., 2020). A recent work has shown that suppression is not uniform and that abundant caveolae are selectively abundant in arteriolar ECs—which facilitates the signaling between neurons, ECs, and vSMCs underlying neurovascular coupling (Chow et al., 2020). Whether this phenomenon varies regionally has not been reported.

#### Mural Cells

Considerable variation is observed in mural cells—a term referring to both vSMCs and PCs in part to account for varying cell identity along the arterial-venous axis (Winkler et al., 2011, 2019; Figures 1B–E). In arteries with diameters > 100 μm, vSMCs are spindle shaped and form concentric rings ∼ 4–10 cells thick (Shiraishi et al., 1986). As arteries progressively branch and taper forming arterioles, vSMC cell layers become thinner and ultimately become discontinuous cells with rod-like or thin lateral processes in more terminal branches (Shiraishi et al., 1986; Iadecola, 2017; Figures 1C,D). In venules and veins, vSMCs remain discontinuous and are stellate in configuration (Hill et al., 2015; Smyth et al., 2018; Vanlandewijck et al., 2018; Figure 1E).

Whether a continuum of mural cell phenotypes exists and the point of transition between vSMC to pericyte remains controversial. Some have suggested that the transition from vSMCs to pericytes is discrete (Hill et al., 2015; Zeisel et al., 2015; Vanlandewijck et al., 2018). Others have described a transitional cell with shared attributes between both cell types (Hartmann et al., 2015; Grant et al., 2019; Grubb et al., 2020; Ratelade et al., 2020). Transitional cells have also been suggested in humans (Smyth et al., 2018; Ratelade et al., 2020). However, classification of these or closely related cells differs between groups with a number of other terms—including ensheathing PCs, pre-capillary PCs, vSMC-PCs hybrids, or precapillary vSMCs (Uemura et al., 2020). Despite these differences, most agree that the contractile cells in pre-capillary arterioles are responsible for regional CBF regulation and neurovascular coupling (Fernandez-Klett et al., 2010; Hill et al., 2015; Kisler et al., 2017b; Hartmann et al., 2020).

Brain PCs also display considerable morphologic heterogeneity—including ensheathing or transitional, mesh, thin-strand, or helical and stellate configurations (Hartmann et al., 2015; Arango-Lievano et al., 2018; Smyth et al., 2018; Grant et al., 2019; Uemura et al., 2020; Figure 1B). Ensheathing PCs—analogous to the transitional cells above—predominately localize to pre-capillary arterioles. Like other PCs, they have an ovoid cell body, but with cell processes that are larger and broader which enwrap and cover much of the endothelial wall (> 90%). They also express contractile proteins—such as smooth muscle α-actin (αSMA) (Hartmann et al., 2015; Smyth et al., 2018; Grant et al., 2019). Contractile protein expression terminates as arterioles branch further to form smaller capillaries. Throughout capillaries and post-capillary venules, PC cell processes assume a mesh-like or thin-strand appearance which covers ∼70 or ∼50% of the endothelial cell well, respectively (Hartmann et al., 2015; Smyth et al., 2018; Grant et al., 2019). The functional significance of these configurations is presently unknown but may reflect a facilitatory role in transport.

PC abundance and coverage of the EC wall is relatively constant in the adult brain—including the cortex, hippocampus, striatum, and cerebellum (Bell et al., 2010; Winkler et al., 2010, 2012). However, PCs are less abundant in the spinal cord and most reduced in the gray matter of the anterior horn (Winkler et al., 2012, 2013). Whether these alterations are the result of the relative abundance or presence of different pericyte subtypes is presently unknown. Much of this work has also been done in rodents and whether species-specific differences as with other cell types exists has also yet to be determined (O’Brown et al., 2018).

#### Perivascular Fibroblast-Like Cells

PVFBs share some cell marker expression with PCs—including N-aminopeptidase (also known as CD13, encoded by *Anpep*) and platelet-derived growth factor receptor-β (*Pdgfr*β) (Soderblom et al., 2013). Unlike PCs, they are found along all vessels expect capillaries (Figures 1B,C,E). Morphologically they are more globular appearance with shorter and finer processes which enwrap the vessel wall. They are also more loosely associated with the vessels—described to be an “awkward hug” (Vanlandewijck et al., 2018; Rajan et al., 2020). Whether different morphologic subtypes exist along the arterial-venous axis or in different brain regions remains to be characterized.

#### Perivascular Macrophages

PVMs are confined to arterioles and venules and are one of several specialized macrophages known as “border associated macrophages”—including leptomeningeal, dural, and choroid plexus macrophages (Goldmann et al., 2016; Utz et al., 2020; Figures 1B,C,E). PVMs adopt a relatively simple ameboid morphology (Zeisel et al., 2015; Kierdorf et al., 2019). They are non-motile, but able to extend cellular processes along the Virchow-Robin Space (Goldmann et al., 2016). Other border associated macrophages are stellate or dendriform appearance (Goldmann et al., 2016; Kierdorf et al., 2019). Whether PVMs has regional or arterial-venous variation in morphology has not been reported.

#### Astrocytes

Morphologic variations in astrocytes have been described for > 100 years and were first introduced by Golgi and Cajal (Garcia-Lopez et al., 2010). Classically astrocytes were divided into two morphologic groups. Protoplasmic astrocytes of the gray matter are highly ramified contacting neuronal cell bodies, dendrites, and the vasculature, whereas fibrous astrocytes characterized as smaller with fewer branches are organized largely around white matter tracts (Oberheim et al., 2006; Miller, 2018; Matias et al., 2019). Between species, protoplasmic astrocytes are larger and extend 10-fold as many cellular processes in humans than rodents (Oberheim et al., 2009). Even greater morphological diversity is observed in different cortical layers (Oberheim et al., 2006, 2009; Lanjakornsiripan et al., 2018). For example, layer 1 astrocytes known as subpial or marginal zone astrocytes display unique molecular expression patterns (Garcia-Marques and Lopez-Mascaraque, 2013; Batiuk et al., 2020). Another subgroup of astrocytes unique to primates—known as interlaminar astrocytes—are located in layer 1 and extend long fibers which extend throughout the cortex and terminate in layers 3/4 (Oberheim et al., 2006, 2009). Extent of ramification or synaptic ensheathment of protoplasmic astrocytes varies between cortical layers (Lanjakornsiripan et al., 2018). In layers 5–6, varicose projection astrocytes also extend long fibers with regularly spaced varicosities (Oberheim et al., 2006, 2009).

With respect to the brain vasculature, astrocyte cell bodies rarely exceed ∼6–10 μm from blood vessels and foot processes form a continuous sheathe around all vessels below the pia—including arterioles, capillaries, and venules (McCaslin et al., 2011). Within the somatosensory cortex, the perivascular astrocytic sheath is thickest for arterioles and thinnest for capillaries. However, capillaries had the greatest density of astrocyte foot processes and separation between astrocytes and capillaries decreased with increasing cortical depth (McCaslin et al., 2011). In other CNS regions, e.g., the retina, astrocytes along veins are large and occur with greater density than arteries (Jammalamadaka et al., 2015). Whether this relationship is maintained in the brain or differs between CNS regions remains to be characterized. During development, the vasculature has been shown to interact with different subtypes of astrocytes or astrocyte-like radial-glial cells depending on location, such as truncated and outer radial glia (Marin-Padilla, 1995; Nowakowski et al., 2016). How different subtypes of astrocytes interact with different aspects of the arterial-venous axis in the adult brain has yet to be completely characterized.

### Transcriptomic Diversification and Subspecialization

Cell diversity in brain vascular cells transcends what can be seen with conventional histologic evaluations. Development of high-throughput single cell transcriptomic technologies identified considerable transcriptional variability of subpopulations within cardinal cell types. Distinct vascular segments have unique functional properties. For example, terminal or pre-capillary arterioles are believed to be the site of regional CBF regulation (Fernandez-Klett et al., 2010; Hill et al., 2015; Kisler et al., 2017b). Capillaries are the site of bidirectional molecular transport (Sweeney et al., 2019). Venules and veins are the site of leukocyte migration and vascular immune responses (Filippi et al., 2018; Lapenna et al., 2018). Dural based sinuses facilitate continued immune surveillance through meningeal lymphatics (Louveau et al., 2015, 2016). In this subsection, we summarize newly defined transcriptional variation and functional distinctions within subpopulations of brain vascular cells (summarized in Table 1).

**TABLE 1:** Enriched cellular markers of transcriptionally-defined subpopulations of brain vascular cells.

Cardinal cell identity	Sub-stratification	Selected enriched markers	References
Endothelial	Arterial	*Gkn3, Hey1, Bmx, Efnb2, Vegfc, Sema3g, Mgp, Fbln2, Fbln5, Cytl1 Tm4sf1*	Sabbagh et al., 2018; Saunders et al., 2018; Vanlandewijck et al., 2018; Zeisel et al., 2018; Kalucka et al., 2020
	Capillary	*Mfsd2a, Tfrc, Meox1, Rgcc*	Sabbagh et al., 2018; Saunders et al., 2018; Vanlandewijck et al., 2018; Zeisel et al., 2018; Kalucka et al., 2020
	Venous	*Slc38a5, Nr2f2, Lcn2, Ccl19, Mafb, Mmm 1, Vwf*	Sabbagh et al., 2018; Saunders et al., 2018; Vanlandewijck et al., 2018; Zeisel et al., 2018; Kalucka et al., 2020
	Tip Cells	*Plaur, Angpt2, Lcp2, Cxcr4, Apln, Kcne3, Mcam, Lamb1, Trp53i11*	Sabbagh et al., 2018
	Choroid plexus	*Plvap, Plpp3, Esm1, Plpp1, Cd24a, Nrp1, Rgcc*	Kalucka et al., 2020
Pericytes		*Abcc9, Kcnj8, Vtn, Ifitm1, Ggt1*	Saunders et al., 2018; Vanlandewijck et al., 2018; Zeisel et al., 2018
Vascular smooth muscle cells	Arterial	*Acta, Tagln, Cnn1, Tinagl1, Fos*	Saunders et al., 2018; Vanlandewijck et al., 2018
	Venous	*Kcnj8, Abcc9, Car4, Slc5a5*	Saunders et al., 2018; Vanlandewijck et al., 2018
Fibroblasts	Perivascular	*Col1a1, Col1a2, Lum, Dcn, Pdgfra*	Saunders et al., 2018; Vanlandewijck et al., 2018;
	Pial	*S100a6, Col18a1, Postn, Lama2, Ngfr*	DeSisto et al., 2020
	Arachnoid	*Ptgds, Aldh1a2, Crabp2, Ogn*	DeSisto et al., 2020
	Dura	*Alpl, Foxc2, Fxyd5, Dkk2, Mgp, Crabp2*	DeSisto et al., 2020
Perivascular macrophages		*Mrc, Lyve1, Lyl1, Spic*	Zeisel et al., 2015

#### Endothelial Cells

EC heterogeneity is observed across organs (Augustin and Koh, 2017). Brain ECs display a number of distinct attributes consistent with their specialized function. For example, brain ECs specifically express a number of transmembrane transporters—such as those involved in the transport of glucose (*Slc2a1*), amino acids (*Slc3a2*, *Slc7a5)*, and fatty acids (*Mfsd2a*) (Sabbagh et al., 2018; Feng et al., 2019; Jambusaria et al., 2020; Kalucka et al., 2020). Brain ECs also demonstrate a unique metabolic gene signature (Kalucka et al., 2020). Others have demonstrated enrichment of translated transcripts specific to neurotransmission, such as synapse organization and neurotransmitter transport (Jambusaria et al., 2020). Enrichment for Wnt signaling and brain EC-specific transcription factors associated with maturation of the BBB are also observed, such as forkhead box F2 (*Foxf2*), forkhead box Q1 (*Foxq1*) or zic family member 3 (*Zic3*) (Hupe et al., 2017; Sabbagh et al., 2018; Kalucka et al., 2020). Brain specific epigenetic regulatory networks, including chromatin accessibility and DNA methylome landscapes, have also been described in brain ECs suggesting added regulatory mechanisms (Sabbagh et al., 2018).

Within the brain, EC gene expression changes along the arterial-venous axis. Capillary and venule ECs preferentially express solute transporters and inflammatory mediators, respectively (Macdonald et al., 2010). scRNAseq studies have demonstrated a transcriptional continuum or zonation characterized by gradual phenotypic changes along the arterial-venous axis. Closer analyses showed that transcription factors were overrepresented in arterial ECs, while transporters were enriched in ECs from capillary and veins. Three additional EC clusters were also identified but were outside the arteriovenous zone and the significance of these were unclear (Vanlandewijck et al., 2018).

scRNAseq technologies have evolved to permit characterization of a greater number of cells, such as droplet based scRNAseq. Studies employing these techniques in the adult murine brain vasculature have similarly showed continuous gene expression changes consistent with smooth EC molecular transitions along the arterial-venous axis (Saunders et al., 2018; Kalucka et al., 2020). A recent study has identified nine distinct EC clusters. Seven correspond to arterial-venous graduation—such as large arteries, arterial shear stress, capillary artery, capillary, capillary venous, and large vein. Two additional clusters were also noted—including choroid plexus ECs and interferon-activated ECs (Kalucka et al., 2020). Others have identified seven distinct subclusters of brain ECs. In addition to observing an arterial-venous continuum, the authors found evidence for further functional subspecialization within subclusters. For example, a subcluster of arterial ECs displayed selective expression of genes implicated in growth factor dependent remodeling, such as matrix gla protein (*Mgp*), fibulin 5 (*Fbln5*), elastin (*Eln*), insulin-like growth factor binding protein 4 (*Igfbp4*), and clusterin (*Clu*). Other processes, such as host immunity or interferon signaling, showed similar expression across vessel types (Saunders et al., 2018).

In the early postnatal brain vasculature, others have demonstrated six EC clusters or subtypes—including arterial, capillary-arterial, capillary-venous, venous, mitotic, and tip cells (Sabbagh et al., 2018). Angiogenic cues specify some endothelial cells to become tip cells—which are motile and able to navigate through tissues. These are followed by less motile stalk ECs which maintain connections with the preexisting vasculature (Hasan et al., 2017; Pitulescu et al., 2017). scRNAseq has shown tip cells are enriched for transcripts encoding membrane or secreted proteins—including ion channels, cell adhesion proteins, receptor tyrosine kinases, extracellular matrix proteins, and other signaling molecules (Sabbagh et al., 2018). Lineage tracing demonstrated that tip cells are more closely related to arterial than venous ECs (Sabbagh et al., 2018). During angiogenesis, proliferating cells have recently been shown to arise from veins (Xu et al., 2014). Consistent with this, shared expression patterns are observed between mitotic, capillary venous, and venous ECs (Sabbagh et al., 2018).

Regional variability has also been reported in the brain EC transcriptome. For example, brain EC expression of transporter genes was upregulated in arterial, capillary, and venous ECs, but not in choroid plexus (Kalucka et al., 2020). Consistent with their fenestrated morphology, there were also selective upregulation in high permeability genes—such as *Plvap* (Kalucka et al., 2020). Other studies have shown similar relative abundances of EC subtypes across 9 distinct brain regions—including the frontal cortex, posterior cortex, hippocampus, striatum, thalamus, globus pallidus externus, and nucleus basalis, subthalamic nucleus, substantia nigra, and ventral tegmental area, and cerebellum (Saunders et al., 2018). Other targeted scRNA approaches geared toward the neuronal-stem cell enriched subventricular zone show EC expression of certain stem cell markers—such as prominin 1 (*Prom1*) and nestin (*Nes*) (Zywitza et al., 2018).

#### Mural Cells

RNAseq experiments have confirmed a number of transcriptomic differences between vSMCs and PCs (Zeisel et al., 2015; He et al., 2016; Chasseigneaux et al., 2018; Saunders et al., 2018; Vanlandewijck et al., 2018). For example, arteriolar vSMCs show enrichment in gene products involved in pathways mediating cell contractility, vascular remodeling, and responses to hypoxia or oxidative stress, whereas mid-capillary PCs are enriched for immune regulatory processes and responses to viruses or toxic substances (Chasseigneaux et al., 2018). Early scRNAseq studies distinguished vSMCs from PCs based on expression of the contractile protein smooth muscle α-actin (*Acta2*) (Zeisel et al., 2015). In contrast to ECs, a more recent scRNAseq study has suggested a punctuated continuum for mural cells with an abrupt transition between arterial/arteriolar vSMCs and PCs. However, PCs appear to form a transcriptional continuum with venous vSMCs (Vanlandewijck et al., 2018). This study also demonstrated differences in expression between arterial and venous vSMCs, such as levels of calponin-1 (*Cnn1)*, *Acta2*, and smooth muscle protein 22-α (*Tagln*) (Vanlandewijck et al., 2018). With different scRNAseq methodologies, others have shown even greater mural size diversity and identified seven distinct subpopulations (Saunders et al., 2018). These approaches have identified specific markers for brain PCs in rodents—such as ATP binding cassette subfamily C member 9 (*Abcc9*), gamma-glutamyltransferase 1 (*Ggt1*), potassium inwardly rectifying channel subfamily J member 8 (*Kcnj8)*, vitronectin (*vtn*) and interferon-induced transmembrane protein 1 (*Ifitm1*) (He et al., 2016; Chasseigneaux et al., 2018; Vanlandewijck et al., 2018), and have found differences in brain PCs in comparison to other organs—such as enrichment in SLC, ABC, and ATP transporters (He et al., 2018; Vanlandewijck et al., 2018).

Many scRNAseq studies have not found transcriptionally distinct brain PC subtypes as supported by morphologic studies (Saunders et al., 2018; Vanlandewijck et al., 2018). A more recent scRNAseq mouse brain atlas has shown three distinct PC populations which vary in relative abundance across brain regions (Zeisel et al., 2018). Techniques—such as fluorescent activated cell sorting—have supported molecularly distinct PC subtypes based on transmembrane protein expression (Park et al., 2016). Others have shown that another subpopulation of brain PCs express the gap junction protein connexin 30 (Mazare et al., 2018). A separate group has classified PCs into two subtypes—type A and type B subtypes—based on expression of *Pdgfrα, Pdgfr*β, *Anpep*, desmin (*Des*) and αSMA (Goritz et al., 2011). Type A PCs have been shown to form scars after spinal cord and/or brain injury (Goritz et al., 2011; Birbrair et al., 2014; Dias et al., 2018). However, some have suggested that this classification are not distinct PC populations, but rather more newly defined PVFBs (Soderblom et al., 2013; Vanlandewijck et al., 2018). These studies support distinct subpopulations of brain pericytes. However, future studies are need to more completely characterize regulatory mechanisms contributing to pericyte diversity.

#### Perivascular Fibroblast-Like Cells

scRNAseq first identified 2 PVFBs subpopulations defined by expression of collagens [e.g., collagen type I alpha 1 chain (*Col1a1*) and collagen type I alpha 2 chain (*Col1a2*)], lumican (*Lum*), decorin (*Dcn*), and *Pdgfr*α (Vanlandewijck et al., 2018). With newer droplet-based scRNAseq, up to 7 distinct subtypes have been identified (Saunders et al., 2018). Two subclusters were enriched in membrane transporters and pumps, while others showed higher levels in collagen genes or different extracellular matrix proteins, angiogenesis, and contraction (Saunders et al., 2018). This suggests possible functional subspecialization which has been shown to vary regionally. PVFBs enriched for membrane transport functions and collagen expression were observed with higher relative abundance in the hippocampus/cortex and basal ganglia/thalamus, respectively (Saunders et al., 2018). Others have shown PVFBs are transcriptionally similar to populations of vascular leptomeningeal cells (pial and arachnoid cells) that reside in the meninges (Marques et al., 2016; DeSisto et al., 2020). Expression of the cytokine interleukin 33 (*Il33*) and the prostaglandin D2 synthetase (*Ptgds*) and markers of pial, arachnoid, and dural meningeal fibroblasts, may also help distinguish between PVFBs subpopulations located in the brain vs. fibroblasts in the meninges (Zeisel et al., 2018; DeSisto et al., 2020). Both *Col1a1*-positive and *Rgs5*-positive populations of mural cells representing PVFBs and PCs, respectively, can sense inflammatory stimuli and signal to the brain through C–C motif chemokine ligand 2 (*Ccl2*) (Duan et al., 2018). Apart from this study, however, the functional significance of this heterogeneity has yet to be defined and future studies are needed.

#### Perivascular Macrophages

PVMs are transcriptionally closely related to microglial, but may be distinguished by expression of mannose receptor C-type 1 (*Mrc*, encodes CD206) and lymphatic vessel endothelial hyaluronan receptor 1 (*Lyve1)* or *Cd36* (Zeisel et al., 2015; Goldmann et al., 2016). Some have shown that expression of LYL1 basic helix-loop-helix family member (*Lyl1*) and Spi-C transcription factor (*Spic*) were specific to PVMs in the brain (Zeisel et al., 2015). Others have distinguished CNS-associated macrophages from peripheral immune cells through an absence of α4-integrin (*Itga4*) and *Cd44* (Butovsky et al., 2012; Ajami et al., 2018; Jordao et al., 2019; Kierdorf et al., 2019).

Profiling of all CNS-associated macrophage populations—perivascular, meningeal, and choroid plexus, demonstrates three transcriptionally distinct clusters which share a core signature consisting of *Mrc1*, platelet factor 4 *(Pf4)*, membrane-spanning four domains subfamily A member 7 (*Ms4a7*), stabilin 1 (*Stab1*), and carbonyl reductase 2 (*Cbr2)* (Jordao et al., 2019). Other distinct PVM subclasses have also been defined in neuroinflammation—such as expression of antigen-presenting MHC class II molecules (Jordao et al., 2019). Other brain inflammatory cells—such as microglia—are transcriptional similar across brain regions in adults but display added heterogeneity in different developmental periods (Li et al., 2019). Whether regional or context-dependent heterogeneity exists specifically within PVMs has yet to be reported.

#### Astrocytes

Astrocyte heterogeneity has been identified both morphologically and transcriptionally (Bayraktar et al., 2014). For example, fibrous astrocytes of the white matter more highly express glial fibrillary acidic protein (Gfap) than protoplasmic astrocytes of cortical gray matter (Cahoy et al., 2008). Early scRNA seq experiments transcriptional defined two separate populations of cortical astrocytes distinguished by expression of glial fibrillary acidic protein (*Gfap*) and milk fat globule-EGF factor 8 protein (*Mfge8*) (Zeisel et al., 2015). A more recent scRNAseq study in the cortex and hippocampus has identified 5 transcriptionally distinct astrocyte subtypes in adult rodents (Batiuk et al., 2020). Each with a unique spatial distribution, and with some exceptions roughly coincide with morphologically defined subtypes (Lanjakornsiripan et al., 2018). Others have similarly supported the presence of five molecularly distinct astrocytes with different techniques—such as fluorescence-activated cell sorting (John Lin et al., 2017).

With inclusion of additional brain regions, others have found seven distinct subtypes of astrocytes with scRNAseq—including Bergmann glia of the cerebellum, olfactory-specific astrocytes, two telencephalon specific astrocytes, two non-telencephalon astrocytes, and a myocilin (*Myoc*) expressing astrocyte of the dorsal midbrain (Zeisel et al., 2018). Expression of *Mfge8* and angiotensinogen (*Agt*) sharply distinguished astrocytes from the telencephalon and diencephalon (Zeisel et al., 2018). Others have defined further regionally distinct astrocyte subtypes in other CNS regions—such as the spinal cord and retina (Hochstim et al., 2008; Menon et al., 2019). Additional astrocytic transcriptional heterogeneity has also been identified within cytoarchitecturally defined brain subregions. For example, laminar spatial gene expression is observed within astrocytes from distinct cortical layers which also varies between functionally distinct regions of cortex (Bayraktar et al., 2020). With emerging sequencing technologies it is therefore likely additional astrocyte subtypes will be defined. How different transcriptional diversity of astrocytes influences vascular interactions has yet to be systematically studied.

## Discussion and Future Directions

Advances in scRNAseq technology have identified heterogeneity within cells comprising the brain vasculature beyond what can be seen with a microscope. The functional significance of transcriptomic variation has been implied based on enriched signaling or ontologic pathways. Future studies with subtype specific enhancer constructs and/or viruses are needed to directly understand the functional delineation of cell subtypes (Blankvoort et al., 2018; Nott et al., 2019). Platforms to survey the function of vascular cells are also lacking. While organoids have rapidly increased in popularity to study neuronal brain cell types, these “mini-brains” have only been vascularized with ECs from other organs to date and further lack blood flow (Khakipoor et al., 2020). Regulatory mechanism responsible for the genesis or maintenance of transcriptomic diversity in the context of brain vascular cells is also presently poorly defined. Only a single report has described epigenetic regulatory mechanisms in brain ECs (Sabbagh et al., 2018). Studies have supported that signaling from neighboring glia and neurons contribute to brain specific transcriptomic networks (Hupe et al., 2017; Sabbagh et al., 2018; Jambusaria et al., 2020; Kalucka et al., 2020). Alterations in blood flow-induced wall shear stress may lead to further transcriptional augmentation and in part help delineate cells along the arterial-venous axis (Masumura et al., 2009; Vatine et al., 2019). However, the relative contributions of flow-mediated biomechanical properties, circulating blood cells, and/or brain parenchyma to these observations is presently unknown.

Much of the variation of brain vasculature has been defined in rodents. Whether further diversity is seen in other species—such as humans—is also presently unknown. A number of studies have described alterations of vascular cells in neurovascular disorders—such as neurodegenerative diseases (Alzheimer’s disease and amyotrophic lateral sclerosis), intracerebral hemorrhage, and vascular malformations (Goritz et al., 2011; Winkler et al., 2011, 2013, 2014b, 2015, 2018; Dias et al., 2018; Montagne et al., 2018; Sweeney et al., 2018; Ratelade et al., 2020). How vascular cell heterogeneity is altered in diseases affecting the cerebrovasculature has yet to be defined, and continued efforts are also needed to create *in vitro* human models which retain vascular cell diversity are needed to facilitate disease modeling.

## Author Contributions

JR, CK, and EW designed the review outline, performed the literature search, and wrote the manuscript. DA, EC, KN, DC, AA, and TN provided the critical reviews, revised the manuscript, and provided relevant edits. All authors contributed to the article and approved the submitted version.

## Conflict of Interest

The authors declare that the research was conducted in the absence of any commercial or financial relationships that could be construed as a potential conflict of interest.
